# The Effects of Vision-Related Aspects on Noise Perception of Wind Turbines in Quiet Areas

**DOI:** 10.3390/ijerph10051681

**Published:** 2013-04-26

**Authors:** Luigi Maffei, Tina Iachini, Massimiliano Masullo, Francesco Aletta, Francesco Sorrentino, Vincenzo Paolo Senese, Francesco Ruotolo

**Affiliations:** 1 Department of Architecture and Industrial Design “L.Vanvitelli”, Second University of Naples, Via San Lorenzo ad Septimum, Aversa 81031, Italy; E-Mails: massimiliano.masullo@unina2.it (M.M.); francesco.aletta@unina2.it (F.A.); sorrentino_francesco@ymail.com (F.S.); 2 Department of Psychology, Second University of Naples, Viale Ellittico 31, Caserta 81100, Italy; E-Mails: santa.iachini@unina2.it (T.I.); vincenzopaolo.senese@unina2.it (V.P.S.); francesco.ruotolo@unina2.it (F.R.)

**Keywords:** wind turbine noise, virtual reality, quiet areas, visual influence

## Abstract

Preserving the soundscape and geographic extension of quiet areas is a great challenge against the wide-spreading of environmental noise. The E.U. Environmental Noise Directive underlines the need to preserve quiet areas as a new aim for the management of noise in European countries. At the same time, due to their low population density, rural areas characterized by suitable wind are considered appropriate locations for installing wind farms. However, despite the fact that wind farms are represented as environmentally friendly projects, these plants are often viewed as visual and audible intruders, that spoil the landscape and generate noise. Even though the correlations are still unclear, it is obvious that visual impacts of wind farms could increase due to their size and coherence with respect to the rural/quiet environment. In this paper, by using the Immersive Virtual Reality technique, some visual and acoustical aspects of the impact of a wind farm on a sample of subjects were assessed and analyzed. The subjects were immersed in a virtual scenario that represented a situation of a typical rural outdoor scenario that they experienced at different distances from the wind turbines. The influence of the number and the colour of wind turbines on global, visual and auditory judgment were investigated. The main results showed that, regarding the number of wind turbines, the visual component has a weak effect on individual reactions, while the colour influences both visual and auditory individual reactions, although in a different way.

## 1. Introduction

Preserving the soundscape and geographic extension of quiet areas is a great challenge against the wide spread of environmental noise. The Environmental Noise Directive [1] points out the need to preserve natural quietness, as a resourceful land protection strategy and as a new aim for the management of noise in European countries. The Directive defines two types of quiet areas: “Quiet area in an agglomeration” (areas exposed to a value of L_den_ or other appropriate noise indicators less than a certain value set by the Member State) and “Quiet area in open country” (areas undisturbed by noise from traffic, industry or recreational activities). Nevertheless threshold values for L_den_, as well as the significance of undisturbed area are still unclear.

Regarding the threshold values for L_den_, it is important to point out that low sound levels do not necessarily correspond to undisturbed areas and that there is enough evidence to discount the “energetic criterion” as the criterion for defining such acoustic areas. Instead, the criterion should be based on which sounds are preferred or wanted in a particular context. According to Brown [2], “quietness” is not always the acoustic characteristic that people desire in such areas, so he suggests changing the terminology to “areas of high acoustic quality”. At the same time, Job [3] showed that it is difficult to find a clear threshold noise level for the assessment of community reaction to noise, because annoyance depends on many features of the noise itself, situational factors and individual variation in sensitivity to noise [3,4,5].

The term “quietness” relates to peace and calmness and is often associated with natural environments: this is what people would call a “tranquil” environment [6]. Usually rural areas are considered to be “tranquil” and restorative places [7]. Brown [2] defines the non-urban or rural domain as the context including landscapes outside of urban areas in which people might rest: coastlines, forests, mountains, countryside and so on. Additionally, he includes the much more extensive rural areas in which the main aim is not recreation, but agriculture or similar: all these areas can express different soundscapes that people enjoy. In general, it is evident that people find scenarios involving naturalistic or rural as more relaxing to listen to and less visually disturbing [8,9,10,11,12,13].

However, due to their low population density, rural areas with suitable wind are considered appropriate locations for installing wind farms (WFs) [14]. Despite the fact that WFs are represented as environmentally friendly projects, wind turbines (WTs) are often viewed as visual and audible intruders that spoil the landscape and generate noise [9]. Furthermore, rural places are often landscapes with high values for recreation and tourism that could decrease with the construction of a wind farm.

WTs produce special audible and inaudible sound contribution [15] in three regions of the spectrum, generating: infrasound, low frequency and high frequency noise, according to several mechanisms [16,17]. For this reason, WTs are barely comparable with other noise sources. It has been shown that, compared with other environmental noise sources (road, rail and aircraft noise), noise annoyance due to WTs appears at lower exposure levels [18,19,20] and, like the other noise sources, it could have adverse effects on health-related quality of life [21,22]. Even though most studies about the effects of WTs on noise annoyance refer to people living in residential areas [19,20,23,24], some evidence indicates that living in a rural landscape rather than in an urbanized area enhances the risk of perceiving WTs’ noise and, furthermore, the risk of annoyance [9]. For example, Fidell and colleagues [25] estimated that the noise level tolerated in non-urban areas compared to residential areas was 7 dB(A) lower. Furthermore, Bakker and colleagues [26] found out that the link between the WF sound levels and annoyance is stronger in quiet areas. For this reason, they suggested that the risk of being disturbed by the WFs’ noise during sleep and/or of being distressed is more pronounced in these areas compared to noisy areas.

According to Pedersen and Halmstad [27], one of the main sources of annoyance in rural areas is represented by the visual aspect of the WTs, especially if there is a large wide-open space. The visual impact of WTs has also been shown to be more pronounced in rural areas when compared to more densely populated areas [28]. In a review about the health effect of WTs, Knopper and Ollson [29] pointed out that annoyance provoked by WTs’ is slightly associated with WTs noise, but strongly related to their visual characteristics, individual attitude to WTs and individual noise sensitivity. The impact of WTs on individuals has recently been studied by Ruotolo and colleagues [30] by directly manipulating the presence of visual and auditory characteristics of a wind farm. They found out that the presence of a visual scenario, as compared to the only availability of auditory stimuli, exerts a negative effect on resource-demanding cognitive tasks, but a positive effect on perceived noise annoyance. This indicates that WFs’ visual features modulate the effects of noise and that WFs’ auditory and visual features are processed by people in close interaction. Therefore, the impact of WTs’ visual aspects on people’s noise annoyance could be enhanced in landscapes where the noise sources are easily visible [24]. 

If we accept that the visual aspect of the WFs plays a relevant role in the perceptual process, colours could represent one of the main factors of influence on the subjective perception of the WFs’ noise. In their study, Menzel and Fastl [31] showed that the levels of perceived loudness of a sport car’s noise varied according to the colour of the car. In the same way, Song *et al.* [32] showed that the degree of road traffic’s noise annoyance was modulated by the simultaneous presentation of different colours on a computer screen. The results showed an interaction between colour perception and noise level between 45 dB and 65 dB. In particular, compared with other colours, red colour increased subjective noise annoyance. Similar results were found by Kim *et al.* [33]. In their experiment, 12 colours were presented on a full screen while a wide range of pink noise was served as the audio components of the stimuli. The sound pressure level of the presented noise was set from 40 to 90 dB(A) by 12.5 dB steps. Results showed that when the colour was close to red or white, the noise was perceived as significantly louder and noisier; when the colour was close to green or blue, the noise was perceived significantly softer and quieter. These studies suggest that the visual characteristics of the environments, such as the colours, modulate the perception of the noise. 

The source-subject distance and the contrast between the WTs and their background of sky were found to be good predictors of the perceived impact of WTs. In their experiments, Bishop and Miller [34], used JPG images as the basis for calculating a *visibility index* based on the size and contrast of the towers. The visibility index considered the absolute colour difference (ΔE*_abs_) in the CIELAB colour model. Likewise, Del Carmen Torres Sibille *et al.* [35] developed an indicator to assess the magnitude of the objective aesthetic impact on the landscape caused by the installation of five WFs. The indicator was supposed to combine measures of visibility, colour, fractality and continuity which could be taken from photographs. 

In sum, most of the previously mentioned studies highlight the importance of considering both visual and auditory aspects of WTs when its impact on people experiencing quiet areas has to be assessed. However, to our knowledge, most of the studies that evaluated the effects of WTs on individuals have been conducted in a unimodal condition in which only the acoustic or the visual stimuli were presented to a group of people. Furthermore, only in few cases the visual impact of an existing or future infrastructure has been assessed by means of real scale 3D graphic reconstructions experienced in virtual reality immersive [30] or non-immersive environments.

In this research, we assess how people perceive auditory and visual characteristics of scenarios reproducing a wind farm with different features. In order to reproduce the conditions in which the wind farm can be experienced in natural contexts as closely as possible, the Immersive Virtual Reality (IVR) technology was used. Although the graphics depicting virtual environments are still far from being fully natural, IVR has several advantages: (1) it allows reproduction in a controlled way the most relevant environmental characteristics; (2) users are surrounded by the virtual environment, which gives the impression of being inside the virtual world; (3) users can interact in real time with the environment. These characteristics give to the people the sensation that the simulated world is perceptually convincing and that it can reproduce what happens in the real life [36]. Recently, several theoretical and applied studies have been carried out to increase the reliability of IVR’s techniques [37,38]. Furthermore, the visual representation has been integrated with auralization systems that are meant to create audible sound files from numerical data, which can be simulated, measured or synthesised, obtaining convincing audio presentations. Maffei *et al.* used the IVR system in different studies [39,40,41]. In a case-study of a new highway [41], it was found that conventional methods to assess noise impact based on noise maps and noise limits did not match even the real response of the population. These results suggested that the application of a methodology which contemplates the audio and visual annoyance reaction of a sample of the population can lead to more information on the noise impact assessment of a new project.

This paper presents a study where, by means of IVR techniques, some visual and acoustical aspects of the impact of a wind farm on a sample of subjects were assessed and analyzed at three different distances from the wind farm (150, 250 and 500 m). The influence of the number of turbines and the colour of turbines on the global, visual and the acoustic judgment was investigated.

## 2. Experimental Section

### 2.1. Preliminary Test

Forty-six adults (34 males), aged 18–33 years (M = 25.33; S.D. = 3.08), participated in the study. The group was selected among the inhabitants living both in urban and in rural/quiet areas. A preliminary survey was submitted to the group. At the beginning, each participant had to fill out a questionnaire regarding age, gender, education, and neighbourhood characteristics. Afterwards, four main statements were submitted to participants:

(S1) “I have been annoyed by noise in the last 12 months”
(S2) “I am in favour of energy coming from green sources”
(S3) “I am in favour of wind farms”
(S4) “Wind farms have a significant impact on landscape”

For every statement, participants had to indicate their agreement by means of a 100-point Likert scale, which ranges from “disagree strongly” to “agree strongly”. The mean results and the standard deviations are reported in Table 1.

**Table 1:** Average and standard deviation for Statements 1 to 4 of the preliminary survey.

	S1	S2	S3	S4
Average	50.5	92.8	74.9	68.7
S.D.	35.2	14.3	26.6	28.5

Afterwards, participants were asked to give judgments on five attributes of the wind farm by answering the following questions: 

“Do you consider wind farm: unpleasant/pleasant (Q1), unnecessary/necessary (Q2), dangerous/safe (Q3), environmentally impacting/environmentally friendly (Q4)”. They could express a score from 0 to 100 (where 0 represents the negative limit and 100 represents the positive limit) on a continuous scale. The mean results and the standard deviations are reported in Table 2.

**Table 2:** Average and standard deviation for Questions 1 to 4 of the preliminary survey.

	Q1	Q2	Q3	Q4
Average	48.5	67.9	66.4	51.7
S.D.	27.9	27.7	28.4	31.7

Considering the personal character of the statements and questions, the great values of S.D. for the preliminary survey are probably due to personal background and/or attitude of the sample towards renewable energy and WFs.

### 2.2. The Audio Signals and the Virtual Scenarios

The sound environment at an existing 16 turbine wind farm located in Frigento (Italy) was recorded by means of a portable M-Audio Microtrack 24/96 device and a set of Sennheiser HDC 451wind-screened binaural headphones. The recordings were carried out at three distances *d_i_* from the boundary of wind farm: *d*_1_ = 150 m, *d*_2_ = 250 m and *d*_3_ = 500 m. At these distances, due to the orography, the recorded acoustic stimuli were representative of three closest wind turbines. These WTs were visible from the recording positions.

The measurements were accomplished by means of a dummy head wearing the headphones and facing perpendicular to the wind turbine at a height of 1.65 m above the ground. For each distance *d_i_* the measurements lasted about 30 min. Afterwards, five sound tracks of 30 s were extracted for each distance. In order to avoid that differences in overall and spectral energetic content, as well as differences in semantic content, could affect the subjective judgments during the tests, a comparison within the five sound tracks for *d*_1_, *d*_2_ and *d*_3_ was carried out. Table 3 shows the mean sound equivalent levels in one-third octave band and the respective standard deviation. Data shows that, for all distances, the standard deviations are less than 1 dB in the frequency range 160–1600 Hz, while outside this range the S.D. are less than 4.5 dB. At the same time the semantic content was evaluated by mean of accurate listening sessions of experts in acoustic.

**Table 3:** Averages and standard deviations one-third octave band Leq at the distance.

Freq.	L_eq,150_	S.D._150_	L_eq,250_	S.D._250_	L_eq,500_	S.D._500_	Freq.	L_eq,150_	S.D._150_	L_eq,250_	S.D._250_	L_eq,500_	S.D._500_
Hz	dB	dB	dB	dB	dB	dB	Hz	dB	dB	dB	dB	dB	dB
31.5	47.8	1.5	49.0	3.2	46.1	3.3	800	30.9	0.4	29.3	0.3	28.7	0.1
40	45.9	0.8	46.0	4.5	43.7	4.0	1,000	30.0	0.9	28.4	0.4	28.0	0.1
50	48.2	0.9	43.7	2.6	41.2	1.3	1,250	28.7	0.4	27.6	0.4	27.6	0.2
63	45.4	1.1	39.2	0.9	39.3	0.6	1,600	27.7	0.1	27.2	0.3	27.3	0.5
80	43.6	4.4	37.7	0.8	38.3	0.6	2,000	27.1	0.4	27.1	0.3	28.8	2.2
100	42.3	1.7	37.6	1.2	38.2	0.4	2,500	30.4	3.5	28.6	1.1	31.2	2.5
125	43.1	0.8	38.6	3.4	41.6	0.9	3,150	30.5	4.5	33.9	2.0	35.0	3.0
160	38.1	1.0	35.4	0.7	35.8	0.3	4,000	28.5	2.5	33.5	2.0	32.5	2.4
200	39.5	0.6	35.8	0.6	34.7	0.3	5,000	26.6	0.2	35.9	2.4	35.2	2.5
250	42.5	0.3	34.0	0.9	33.3	0.3	6,300	26.9	0.1	34.0	3.0	33.5	3.1
315	38.3	0.4	33.3	0.7	32.5	0.6	8,000	27.7	0.1	31.7	1.0	30.8	2.0
400	36.9	0.4	33.0	0.7	32.8	0.3	10,000	28.9	0.4	32.3	2.3	29.9	0.5
500	37.8	0.8	31.3	0.5	31.6	0.1	12,500	39.1	2.0	36.1	1.5	35.0	3.1
630	33.4	0.4	30.2	0.4	29.6	0.1	16,000	51.6	0.3	42.6	0.9	46.4	1.9

**Figure 1:**
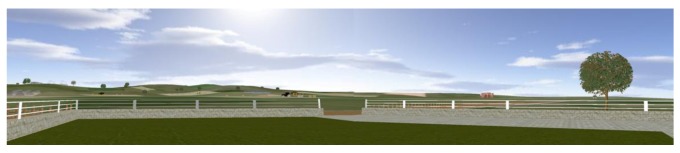
The virtual scenario without wind turbines.

Afterwards, the 3D model of the wind farm was created, also considering the orography of the ground. The cartography and the model of the ground were processed by means of a Computer Aided Design application; the WTs (*h_hub_* = 70 m, *d_rotor_* = 54 m) and all other elements were modeled and textured in 3ds Max. Some elements were collocated in the distance, such as greenhouses, a tractor, and a path. Inside this model a typical rural outdoor scenario was introduced at each distance *d_i_*; it consisted of a courtyard limited by a fence, a table and some benches. The observer was supposed to be seated at a given position inside the courtyard, facing the wind farm. Finally, both the auditory and visual components of the scenarios were uploaded to a Virtual Reality software package (Figure 1).

### 2.3. Experimental Design

Three main factors were manipulated in this experiment: the first factor was the distance from the wind farm (*Distance*), the second factor was the number of wind turbines (*Number*) and the third factor was the colour of the pylons’ bases and of any stripes on the blades of the WTs (*Colour*). The *Distance* factor had three levels: 150, 250 and 500 m. The *Number* factor had three levels: 1, 3 and 6 WTs. The *Colour* factor had four levels: white, red, brown and green.

**Figure 2:**
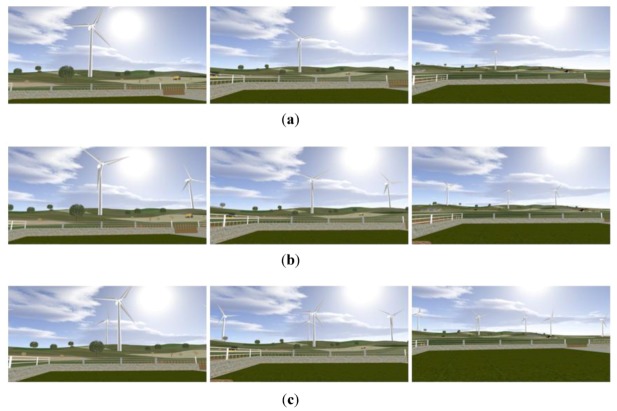
Frames of the scenarios at the distance of 150, 250 and 500 m with one turbine (**a**), three turbines (**b**) and six turbines (**c**).

In order to observe possible interactions between the *Distance* and *Number* factors, all their levels’ combinations were presented to the subjects and a 3 × 3 within subject factorial design was scheduled for the experiment by fixing the *Colour* factor at the white level. In Figure 2 the frames of the nine scenarios are shown (the single frame does not cover all the FOV of the subjects). 

**Figure 3:**
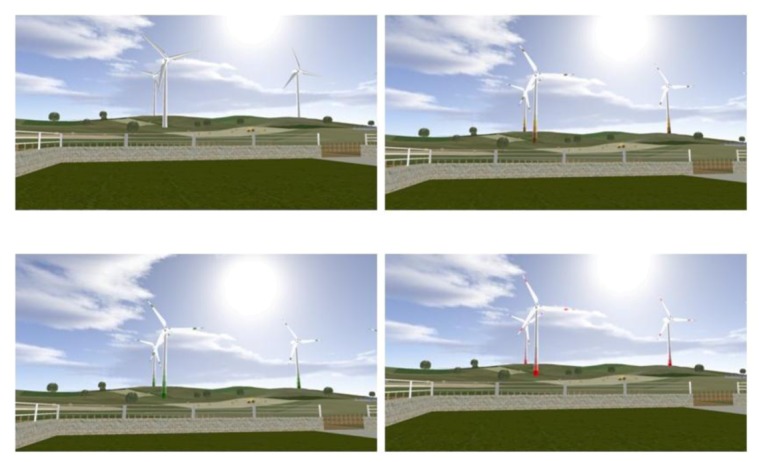
Frames of the scenarios with six turbines at 250 m: white, brown, green and red.

Subsequently, in order to observe the influence of the *Colour* effect on the test, all levels of this factor were presented by fixing the other two factors *Distance* and *Number*, at 250 m level and six wind turbines level, respectively, in a 4-level within subject factor design (Figure 3).

### 2.4. Equipments

The experiment was carried out in a 5 × 5 × 5 m anechoic chamber, where a couple of loudspeakers, a sub-woofer and a Motu 828 Mk3 sound card were used for the playback. The loudspeakers/subwoofer were preferred to the headphones in order to reproduce as best as possible the sound contribution at low frequencies of the WTs. Furthermore, by means of a Cortex Manikin and a CAL01 01dB Metravib (94 dB/1 kHz) calibrator the playback system was calibrated and equalized to reproduce the same auditory conditions and the best fit of the spectrum at the listener’s ear. Moreover, for the test session, the room was equipped with a workstation and the virtual scenarios were presented through a IVR system implemented by an eMagin Z800 3D Visor. The visor presented stereoscopic images at 800 × 600 resolution, refreshed at 60 Hz. Graphics were rendered by a Intel(R) Core(TM) i7 CPU 3.07 GHz processor with a Nvidia GeForce GTX480 graphics card using the software WorldViz 4.0. Head movements (orientation and position) were tracked using the Polhemus Patriot six d.o.f. motion tracking system. 

### 2.5. The Test

All the subjects of the preliminary test were invited to participate in the experiment and took part in an opening session where a tonal audiometric test (500 Hz–4.0 kHz) was presented in order to check their regular hearing. No significant auditory deficiencies were reported and all participants were eligible for and participated in the IVR session. Subsequently, the IVR session started: the subject was given the 3D Visor in order to experience the virtual scenarios and a questionnaire form to mark his answers. Moreover an explanation about the significance of each specific question was given to the subjects. The subject could experience each of the 13 scenarios (3 × 3 + 4 × 1), as long as he wanted. The scenarios were presented in a balanced sequence, in order to minimize the effects that could be generated by the order of the presentation of the scenarios to the subjects. After each scenario the subject was asked to answer the following questions; each question was associated with a dependent variable, according to the following scheme, “Question” (*Dependent variable*):

(1) “How would you rate this scenario?” (*General Evaluation*)
(2) “What do you think about the integration of the wind farm with the landscape?” (*Integration*)
(3) “How would you rate the sound of this scenario?” (*Sound Pleasantness*)
(4) “How loud did you perceive the sound of this scenario?” (*Perceived Loudness*)
(5) “How annoying would you rate this scenario?” (*Noise Annoyance*)
(6) “How stressful would you rate the sound of wind turbines?” (*Sound Stress*)
(7) “How tranquil would you rate the sound of wind turbines?” (*Sound Tranquillity*)
(8) “How visually pleasant would you rate this scenario?” (*General Visual Pleasantness*)
(9) “How visually pleasant would you rate the wind turbines?” (*Wind Turbines Visual Pleasantness*)

For every question, participants could express a score from 0 to 100 on a continuous scale.

## 3. Results

To investigate the effects of the distance from the noise source and the quantity of turbines on the subjective perception of the simulated scenarios, nine 3 × 3 within-subject ANOVAs, that treated the *Distance* (150, 250 and 500 m) and the *Number* of WTs (one, three and six) as 3-level within-subject factors, were performed. As dependent variables we used the nine subjective evaluations considered: General Evaluation, Integration, Sound Pleasantness, Perceived Loudness, Noise Annoyance, Sound stress, Sound Tranquillity, General Visual Pleasantness, and WTs Visual Pleasantness. 

To investigate the effects of WTs’ colour on the subjective perception of the simulated scenarios, nine one-way within subject ANOVAs, that treated the *Colour* (green, white, brown and red) as a 4-level within-subject factor, were performed. As dependent variables we used the same nine subjective evaluations previously considered. In all ANOVA analyses, the Bonferroni correction was used to analyze *post hoc* effects and the magnitude of the significant effects was indicated by partial eta squared (*η*^2^_p_). In all analyses an *alpha* value of 0.05 was used as the criterion to determine significant differences. The mean values are analysed as a function of the *Distance* (see Table 4), the *Number* (see Table 5) and the *Colour* (see Table 6) effect.

**Table 4:** Mean values (standard error) and effect size as a function of the *Distance*.

Variable	Distance			*η* ^2^_p_
	150 m	250 m	500 m	
General Evaluation	41.3_a _(3.2)	54.8_b _(3.2)	63.6_c _(2.8)	0.43 ***
Integration	36.2_a_ (2.9)	48.7_b_ (3.0)	56.9_c_ (3.3)	0.48 ***
Sound Pleasantness	40.0_a_ (2.9)	57.2_b_ (3.1)	62.6_c_ (2.5)	0.47 ***
Perceived Loudness	60.4_a_ (3.2)	42.3_b_ (3.0)	38.3_b_ (2.5)	0.52 ***
Noise Annoyance	55.8_a_ (3.1)	35.7_b_ (3.1)	32.3_b_ (2.7)	0.50 ***
Sound Stress	64.1_a_ (2.6)	46.6_b_ (2.5)	43.4_b_ (2.6)	0.50 ***
Sound Tranquillity	34.1_a_ (2.6)	54.3_b_ (3.2)	60.0_c_ (2.8)	0.52 ***
General Visual Pleasantness	41.9_a_ (2.7)	54.6_b_ (2.8)	58.5_b_ (2.5)	0.36 ***
WTs Visual pleasantness	29.1_a_ (3.2)	45.9_b_ (3.7)	49.7_b_ (3.4)	0.54 ***

Note: For each variable, equal letters indicate equal means (*p* > 0.05, Bonferroni adjusted); *η*^2^_p_ = partial eta squared; *** *p* < 0.001.

**Table 5:** Mean values (standard error) and effect size as a function of the *Number* of turbines.

Variable	Number of turbines			*η* ^2^_p_
	1	3	6	
General Evaluation	57.3_a_ (3.2)	51.6_a,b_ (2.7)	50.8_b_ (3.1)	0.09 *
Integration	50.1_a_ (3.1)	46.3_a_ (2.8)	45.4_a_ (3.1)	0.06
Sound Pleasantness	55.8_a_ (2.4)	52.9_a,b_ (2.9)	51.1_b_ (2.4)	0.08 *
Perceived Loudness	45.7_a_ (2.6)	47.0_a_ (3.0)	48.3_a_ (2.8)	0.02
Noise Annoyance	38.9_a_ (2.5)	41.1_a,b_ (3.0)	43.8_b_ (2.6)	0.08 *
Sound Stress	51.4_a_ (2.2)	50.8_a_ (2.8)	51.9_a_(2.2)	0.0
Sound Tranquillity	50.7_a_ (2.1)	49.6_a_ (3.0)	48.0_a_ (2.8)	0.02
General Visual Pleasantness	51.3_a_ (2.7)	54.2_a_ (2.5)	49.5_a_ (2.6)	0.05
WTs Visual pleasantness	42.0_a_ (3.4)	43.6_a_ (3.2)	39.1_a_ (3.5)	0.07

Note: For each variable, equal letters indicate equal means (*p* > 0.05, Bonferroni adjusted); *η*^2^_p_ = partial eta squared; * *p* < 0.05.

**Table 6:** Mean values (standard error) and effect size as a function of the *Colour* of turbines.

Variable	Colour of turbines				*η* ^2^_p_
	Green	White	Brown	Red	
General Evaluation	59.4_a_ (4.1)	52.2_a,b_ (3.9)	45.0_b,c_ (4.2)	40.9_c_ (4.3)	0.14 ***
Integration	55.4_a_ (4.2)	45.8_b_ (3.6)	39.6_b,c_ (4.0)	34.0_c_ (4.0)	0.18 ***
Sound Pleasantness	56.5_a_ (3.4)	56.6_a_ (3.6)	52.2_a,b_ (3.7)	47.6_b_ (3.4)	0.07 *
Perceived Loudness	44.8_a,b_ (3.6)	42.5_a_ (3.8)	48.6_a,b_ (3.7)	51.8_b_ (3.7)	0.07 *
Noise Annoyance	38.7_a_ (3.5)	39.0_a_ (3.7)	45.3_a_ (3.5)	45.2_a_ (3.8)	0.05
Sound Stress	46.0_a_ (3.5)	47.2_a_ (3.2)	51.7_a,b_ (3.3)	54.0_b_ (3.3)	0.06 *
Sound Tranquillity	54.2_a,b_ (3.8)	55.1_a_ (4.1)	50.1_a,b_ (3.9)	45.2_b_ (3.4)	0.06 *
General Visual Pleasantness	58.9_a_ (3.8)	52.0_a,b_ (3.2)	47.0_b,c_ (4.0)	41.4_c_ (3.8)	0.14 ***
WTs Visual pleasantness	48.6_a_ (4.5)	45.0_a_ (3.9)	39.4_a,b_ (4.9)	31.9_b_ (4.4)	0.12 ***

Note: For each variable, equal letters indicate equal means (*p* > 0.05, Bonferroni adjusted); *η*^2^_p_ = partial eta squared; * *p* < 0.05; *** *p* < 0.001.

### 3.1. Analyses on the General Evaluation

The ANOVA on the General Evaluation showed that subjective ratings were influenced by the *Distance* from the noise source, *F*(2, 90) = 33.987, *p* < 0.001, *η*^2^_p_ = 0.430, and by the *Number* of WTs, *F*(2, 90) = 4.605, *p* = 0.012, *η*^2^_p_ = 0.093. The *Distance* × *Number* interaction was not significant, *F*(4, 180) = 1.558, p = 0.187, *η*^2^_p_ = 0.033. Regarding the *Distance* main effect, the mean comparison showed that when participants were placed 500 m from the noise source, the scenario was rated as more pleasant compared to both the 250 m and the 150 m scenarios. Moreover, a significant difference was observed between the latter two scenarios (see Table 4).

As regards the *Number* main effect, the mean comparison showed that participants rated the scenario with one turbine as more pleasant than the six turbines scenario. The differences between the three turbines scenario and the others were not significant (see Table 5).

The ANOVA that evaluated the *Colour* effect showed that the General Evaluation was influenced by this factor, *F*(3, 135) = 7.326, *p* < 0.001, *η*^2^_p_ = 0.140. Participants rated the scenarios with green and white turbines as more pleasant than the brown and red turbines scenarios (see Table 6).

### 3.2. Analyses on Integration

The ANOVA on the evaluation of the general Integration between the WTs and the environment showed that subjective ratings were influenced by the *Distance*, *F*(2, 88) = 39.99, *p* < 0.001, *η*^2^_p_ = 0.476. Neither the *Number* main effect, *F*(2, 88) = 2.91, *p* = 0.060, *η*^2^_p_ = 0.062, nor the *Distance* × *Number* interaction were significant, *F*(4, 176) = 2.11, *p* = 0.081, *η*^2^_p_ = 0.046. Regarding the *Distance* main effect, the mean comparison showed that participants rated the 500 m scenario as the more integrated scenario in respect to both the 250 m and the 150 m scenarios. Moreover, a significant difference was observed between the latter two scenarios (see Table 4).

The ANOVA that evaluated the *Colour* effect showed that the general Integration evaluation was influenced by this factor, *F*(3, 135) = 9.685, *p* < 0.001, *η*^2^_p_ = 0.177. Participants rated the scenario with green turbines as the most integrated with respect to white, brown and red turbines scenarios. Moreover, the scenario with red turbines was considered the less integrated (see Table 6).

### 3.3. Analyses on Sound Pleasantness

The ANOVA on the Sound Pleasantness evaluations showed that subjective ratings were influenced by the *Distance*, *F*(2, 90) = 39.270, *p* < 0.001, *η*^2^_p_ = 0.466, and by the *Number* of WFs, *F*(2, 90) = 4.046, *p* = 0.021, *η*^2^_p_ = 0.082. The *Distance* *Number* interaction was not significant, *F*(4, 180) = 2.011, *p* = 0.095, *η*^2^_p_ = 0.043. As regards the *Distance* main effect, the mean comparison showed that participants rated the 500 m scenario as the more pleasant with respect to both the 250 m and the 150 m ones. Moreover, a significant difference was observed between the latter two scenarios (see Table 4). With regards to the *Number* main effect, the mean comparison showed that participants rated the scenario with one turbine as more pleasant than the six turbines scenario. The differences between the three turbines scenario and the others were not significant (see Table 5).

The ANOVA that evaluated the *Colour* effect showed that the Sound Pleasantness evaluation was influenced by this factor, *F*(3, 135) = 3.377, *p* = 0.020, *η*^2^_p_ = 0.070. Participants rated the scenario with green and white turbines as more pleasant than the red turbine scenario. No differences were observed between the brown turbine scenario and the others (see Table 6).

### 3.4. Analyses on Perceived Loudness

The ANOVA on the Perceived Loudness showed that subjective ratings were influenced exclusively by the *Distance*, *F*(2, 90) = 47.871, *p* < 0.001, *η*^2^_p_ = 0.515. Neither the *Number* main effect, *F*(2, 90) = 0.866, *p* = 0.424, *η*^2^_p_ = 0.019, nor the *Distance* × *Number* interaction were significant, *F*(4, 180) = 0.452, *p* = 0.771, *η*^2^_p_ = 0.010. Regarding the *Distance* main effect, the mean comparison showed that participants rated the 150 m scenario as the scenario associated with the loudest sound (*M* = 60.4, *p*s < 0.05) in respect to both the 250 m and the 500 m scenarios. The difference between the latter two scenarios was not significant (see Table 4).

The ANOVA that evaluated the *Colour* effect showed that the Perceived Loudness evaluation was influenced by this factor, *F*(3, 135) = 3.186, *p* = 0.026, *η*^2^_p_ = 0.066. Participants rated sounds of the red turbines scenario as louder than sounds of the white turbines scenario. No differences were observed in the comparison between white or red turbines scenarios and the others (see Table 6).

### 3.5. Analyses on Noise Annoyance

The ANOVA on the Noise Annoyance evaluations showed that subjective ratings were influenced by the *Distance* from the noise source, *F*(2, 90) = 45.596, *p* < 0.001, *η*^2^_p_ = 0.503, and by the *Number* of WFs, *F*(2, 90) = 3.852, *p* = 0.025, *η*^2^_p_ = 0.079. The *Distance* × *Number* interaction was not significant, *F*(4, 180) = 1.336, *p* = 0.258, *η*^2^_p_ = 0.029. With regards to the *Distance* main effect, the mean comparison showed that participants rated the 150 m scenario as the more annoying with respect to both the 250 m and the 500 m scenarios. The difference between the latter two scenarios was not significant (see Table 4). Regarding the *Number* main effect, the mean comparison showed that participants rated the scenario with six turbines as more annoying than the one turbine scenario. The differences between the three turbines scenario and the others were not significant (see Table 5).

The ANOVA that evaluated the *Colour* effect showed that the Noise Annoyance evaluation was not influenced by this factor, *F*(3, 135) = 2.268, *p* = 0.084, *η*^2^_p_ = 0.048*.*

### 3.6. Analyses on Sound Stress

Regarding the evaluation of the perceived stress associated with the sounds, the ANOVA showed that subjective ratings were influenced only by the *Distance*, *F*(2, 90) = 44.306, *p* < 0.001, *η*^2^_p_ = 0.496*. * Neither the *Number* main effect, *F*(2, 90) = 0.176, *p* = 0.839, *η*^2^_p_ = 0.004*,* nor the *Distance* × *Number* interaction were significant, *F*(4, 180) = 1.630, *p* = 0.168, *η*^2^_p_ = 0.035. With regards to the *Distance* main effect, the mean comparison showed that participants rated the 150 m scenario as the most stressing scenario in respect to both the 250 m and the 500 m scenarios. Not significant was the difference between the latter two scenarios (see Table 4).

The ANOVA that evaluated the *Colour* effect showed that the Sound Stress evaluation was influenced by this factor, *F*(3, 135) = 2.905, *p* = 0.037, *η*^2^_p_ = 0.061. Participants rated the red turbines scenario as more stressing than the green and the white turbines scenarios. No differences were observed between the brown turbines scenario and the others (see Table 6).

### 3.7. Analyses on Sound Tranquillity

The ANOVA on the Sound Tranquillity evaluations showed that subjective ratings were influenced only by the *Distance*, *F*(2, 30) = 48.823, *p* < 0.001, *η*^2^_p_ = 0.520. Neither the *Number* main effect, *F*(2, 30) = 0.852, *p* = 0.430, *η*^2^_p_ = 0.019*,* nor the *Distance* × *Number* interaction were significant, *F*(4, 60) = 2.363, *p* = 0.055, *η*^2^_p_ = 0.050. With regards to the *Distance* main effect, the mean comparison showed that participants rated the 500 m scenario as the more tranquil scenario in respect to both the 250 m and the 150 m scenarios. Moreover, a significant difference was observed between the latter two scenarios (see Table 4).

The ANOVA that evaluated the *Colour* effect showed that the Sound Tranquillity evaluation was influenced by this factor, *F*(3, 135) = 2.823, *p* = 0.041, *η*^2^_p_ = 0.059. Participants rated sounds of the red turbines scenario as less tranquil than sounds of the white turbines scenario. No differences were observed in the comparison between the white or the red turbines scenarios and the others (see Table 6).

### 3.8. Analyses on General Visual Pleasantness

The ANOVA on the General Visual Pleasantness showed that subjective ratings were influenced only by the *Distance*, *F*(2, 90) = 25.274, *p* < 0.001, *η*^2^_p_ = 0.360. Neither the *Number* main effect, *F*(2, 90) = 2.413, *p* = 0.095, *η*^2^_p_= 0.051, nor the *Distance* × *Number* interaction were significant, *F*(4, 180) = 0.336, *p* = 0.854, *η*^2^_p_= 0.007. Regarding the *Distance* main effect, the mean comparison showed that participants rated the 150 m scenario as the less visually pleasant scenario in respect to both the 250 m and the 500 m scenarios. The difference between the latter two scenarios was not significant (see Table 4).

The ANOVA that evaluated the *Colour* effect showed that the General Visual Pleasantness evaluation was influenced by this factor, *F*(3, 135) = 7.477, *p* < 0.001, *η*^2^_p_= 0.142. Participants rated the red turbines scenario as less pleasant than the green and the white turbines scenarios. The brown turbines scenario was rated as less pleasant than the green one, while no significant differences were observed between the brown turbines scenario and both the white and the red turbines scenarios (see Table 6).

### 3.9. Analyses on WTs Visual Pleasantness

The ANOVA on the WTs Visual pleasantness showed that subjective ratings were influenced only by the *Distance*, *F*(2, 70) = 41.302, *p* < 0.001, *η*^2^_p_ = 0.541. Neither the *Number* main effect, *F*(2, 70) = 2.622, *p* = 0.080; *η*^2^_p_= 0.070, nor the *Distance* × *Number* interaction were significant, *F*(4, 140) = 0.284; *p* = 0.888; *η*^2^_p_= 0.008. Regarding the *Distance* main effect, the mean comparison showed that participants rated the 150 m scenario as the less visually pleasant scenario in respect to both the 250 m and the 500 m scenarios. The difference between the latter two scenarios was not significant (see Table 4).

The ANOVA that evaluated the *Colour* effect showed that the WTs Visual pleasantness evaluation was influenced by this factor, *F*(3, 123) = 5.486; *p* < 0.001; *η*^2^_p_= 0.118. Participants rated the red turbines scenario as less pleasant than the green and the white turbines scenarios. No differences were observed between the brown turbines scenario and the others (see Table 6).

## 4. Conclusions

The main goal of this study was to evaluate the effects of some visual and acoustical aspects of the impact of a wind farm in a quiet area. In order to achieve this goal, an innovative multisensory methodology that integrates auditory and visual components was used. By means of Immersive Virtual Reality equipment different wind farm configurations were created in a 1:1 scale scenario and presented to individuals. The virtual models were created to evaluate the effects of three different components that according to the literature are known to affect individual reactions: (a) the *Distance* from WTs and noise; (b) the *Number* of WTs and noise sources; and (c) the *Colour* of WTs. In the virtual presentation, participants perceived both the visual and the auditory stimuli from the wind farm. They were surrounded by a typical rural outdoor environment and could interact actively with it in a real time. In this way the sense of “being there” was stimulated. After the presentation of each scenario, participants were asked to answer to a series of questions designed to evaluate the effect of the wind farm on the perception of the environment, the integration of the new infrastructure into the existing environment, and both auditory and visual subjective effects. The IVR approach was preferred because it allowed manipulating the considered factors in a controlled way, in order to dissociate the auditory and visual features of each investigated scenario. In our study a set of auditory stimuli, representative of the auditory scenarios at three distances, were associated with different visual stimuli to evaluate the contribution of the *Number* and *Colour* factors on the subjective perception.

As expected, the results showed that the factor Distance from the WF is the stronger factor that affects individual reactions to the WFs. Indeed, for all the considered variables, the effect was significant and large. In particular the data showed that the more distance from the WF, the more positive is the general evaluation of the scenario or the integration of the WF into the existing environment. Moreover, the more distance from the WF, the less is the Perceived Loudness, the Noise Annoyance and the stress caused by wind turbines’ sound, and the more positive is the perceived visual pleasantness. These results are in line with the extensive literature showing that the source-subject distance is an important factor that determines the magnitude of the WFs’ impact on the individual [34].

Regarding the manipulation of the *Number* factor, the results showed that this visual component has a weak effect on individual reactions. Data showed that this factor affects the General Evaluation of the scenario and the perceived Sound Pleasantness and Noise Annoyance. The effects were significant but small. In particular, data showed that: the higher the number of wind turbines is, the less the evaluation of audio-visual pleasantness of the environment is and the more the Noise Annoyance is. No significant differences were observed as regards Perceived Loudness, Sound Stress, Sound Tranquillity and the visual pleasantness. It is important to highlight that the auditory stimuli considered for this comparison belonged to the same groups, with similar spectral levels and semantic contents. For this reason, from one side we can say that the absence of any difference associated with the sound dimensions was expected, but on the other side we can confirm that the observed differences are ascribable mostly to the visual component with respect to the auditory ones.

Finally, regarding the manipulation of WTs’ colour, the results showed that colour influences both visual and auditory individual reactions, although in a different way. It has a large effect on the visual evaluations and a medium effect on the auditory evaluations. This latter effect is not trivial and confirmed the relevance of the visual components on the auditory evaluations and their integration. More in detail, data showed that over and above the auditory parameters, green and white turbines are preferred to brown and red ones in terms of general evaluation, integration, Sound Pleasantness, loudness, annoyance and visual pleasantness. Moreover, a slight preference was observed for green turbines that are evaluated as the most integrated. These results further confirm the interconnectedness between the auditory and visual components and that the presence of specific contextual visual information associated with sound source influences individual perceptions in a broad way [28,31,32,33,34,35].

Overall, it is important to point out that the results of this study are in line with the existing field studies and confirm that the innovative multisensory Immersive Virtual Reality methodology adopted here represents a suitable tool for the Environment Impact Assessment [30,37,38,39,40,41]. The proposed methodology seems to allow the investigation of the potential effects of the introduction of new infrastructure in an existing environment and to define in advance the better project choices that facilitate the preservation of both acoustic and visual quality of the environment [8,9,10,11,12,13]. However, extended experimental sessions are needed to make more robust its ecological validity, particularly by comparing the results of *in situ* evaluations.
